# Editorial: Decoding checkpoint inhibitor-induced immune-related adverse events, volume II

**DOI:** 10.3389/fendo.2023.1317220

**Published:** 2023-10-19

**Authors:** Deborah L. Burnett, Megan B. Barnet, Ania Moxon, Venessa Tsang, Katherine Samaras

**Affiliations:** ^1^ Immunology Division, Garvan Institute of Medical Research, Darlinghurst, NSW, Australia; ^2^ School of Clinical Medicine, Faculty of Medicine and Health, University of New South Wales (UNSW), Sydney, NSW, Australia; ^3^ Department of Medical Oncology, St Vincent’s Hospital, Darlinghurst, NSW, Australia; ^4^ Faculty of Medicine and Health, University of Sydney, Sydney, NSW, Australia; ^5^ Department of Endocrinology, Royal North Shore Hospital, St Leonards, NSW, Australia; ^6^ Department of Endocrinology, St Vincent’s Hospital, Darlinghurst, NSW, Australia; ^7^ Clinical Obesity, Nutrition, and Adipose Biology Lab, Clinical Science Pillar, Garvan Institute of Medical Research, Darlinghurst, NSW, Australia

**Keywords:** immune related adverse effects, immune check inhibitor (ICI), PD-1 - PD-L1 axis, CTLA -4, cancer immunotherapy

Immune checkpoint inhibitors (ICIs), particularly those targeting the Programmed Death 1 (PD-1) or Cytotoxic T-Lymphocyte Associated 4 (CTLA-4) axes have redefined cancer treatment outcomes. The capacity of these therapies to redirect the immune system is linked to off-target toxicities. These immune-related adverse events (irAEs) exhibit varied clinicopathological features. This Research Topic builds on our previous topic focusing specifically on ICI-induced Endocrinopathies (1) and extends into a wider range of immune toxicities. This Research Topic incorporates disciplines including endocrinology, immunology, oncology, gastroenterology, diagnostic radiography and epidemiology to help guide clinical practice and research directions.

The topic is framed through a bibliometric analysis by Jiang et al. which charts the field’s evolution over 2005-2022. Initially slow, interest in the area surged from 2015. irAE papers were published in both cancer and immunology journals, utilising a variety of keywords, highlighting a challenge for researchers seeking information on the topic.

Anpalakhan et al. presented a sub-analysis of the Spinnaker study, a retrospective multicentre observational study exploring patients with non-small-cell lung cancer receiving pembrolizumab with platinum-based chemotherapy (2). The study reported that 43% experienced irAE: two-thirds Grade 1–2, one-third Grade 3–4. The study replicated what has been observed in other studies: that the development of irAEs portends a better intervention response. Further research in immune markers of irAEs for early intervention and response is required.

This Research Topic also delves, through the work of Zhao et al., to focus on pancreatitis; a rare but significant irAE. The team analysed over 40,000 patients in 59 randomized controlled trials (RCTs), reporting an incidence of pancreatitis of 0.93% for single-agent ICI, and 1.1% for combination blockade. The group found patients treated with immunotherapy for melanoma had the highest incidence of amylase/lipase elevation, and that pancreatitis was more common with PD-1 compared with PD-L1 blockade. This review adds relevant insight to understanding which populations might benefit from proactive pancreatitis monitoring.

The latest evidence on PD-1 inhibitor-associated gastrointestinal toxicity is reviewed by Cheng et al., including clinical manifestations, grading, mechanisms, treatments, biomarkers, and risk-stratification. This comprehensive review raises the importance of PD-1 rechallenge after ICI-related colitis, which can lead to colonic perforation. The review highlights the need for further research on coliits indicators to clarify the risk benefit balance in this setting.

This Research Topic also incorporates studies focusing on improved strategies for monitoring and predicting irAEs.

Baier et al. describe PD-L1 upregulation in intra-renal and urinary kidney cells as a pathology biomarker. They propose evaluation of urinary PD-L1-positive kidney cells for biomonitoring for ICI-related nephrotoxicity. The study sets precedent for exploring non-conventional (but clinically-feasible) monitoring techniques to improve diagnosis and irAE outcomes.

Huang et al. reviewed specific features of ICI-related pneumonitis by computed tomography (CT). They emphasise the potential of CT radiomics to distinguish ICI-related pneumonitis (CIP) compared to pneumonitis induced by radiation. This review highlights the high-accuracy of radiomics models to predict the development of CIP from CT images and the potential these tools hold for pneumonitis management. Huang et al. noted the challenges of machine standardisation and stress the need for larger comparative studies in the field.

Advances in theranostics facilitate identification of tissue infiltrating CD8+ T-cells using PET imaging. Bol et al. present a melanoma patient who developed ICI-related hypophysitis. Imaging detected increased pituitary CD8+ infiltration, with concomitant tracer uptake in known cerebral metastasises, indicating ICI-induced CD8+ tumor infiltration. These findings support the role of CD8+ T-cells in the tumouricidal effects of ICI, and their role in irAEs, although further validation from clinical trials is required.

Zhang et al. sourced irAE reports from the US Food and Drug Administration’s Adverse Event Reporting System to establish improved predictive irAE models. They linked these reports with data across 22 cancer types, uncovering key factors linked to irAE frequency. These factors included the tumour mutational burden (TMB), immune composition and expression signatures and transcriptional expression of checkpoint molecules. This study built composite models combining TMB, naïve CD4+ T cells and dendritic cells which displayed high predictive accuracy for irAE occurrences. Zhang et al. also utilised mRNA expression to develop gene-based predictive models. Interestingly, many of the factors they identified lack known associations with ICI efficacy, underscoring the significance of irAE-specific correlation models.

This Research Topic also incorporated studies exploring irAEs from less well-established ICI therapies. This includes combination regimes involving taxane-based chemotherapeutic nanoparticles, such as nab-paclitaxel (nab-PTX) and PTX. Hao et al. conducted a meta-analysis, encompassing 22 published RCTs with 15962 patients, to assess the risk of irAEs when comparing ICI monotherapy to combination therapy with nab-PTX/PTX. Their findings suggested that combination therapy reduced the risk of specific irAEs, particularly those related to thyroid dysfunction or pneumonitis. However, the impact of combination therapy on other irAEs was less conclusive. The underlying mechanism and potential confounding factors, such as corticosteroid pre-treatment in PTX chemotherapy, warrant further investigation.

The topic also serves as a focus point for unusual presentations of irAEs.

This includes a case report by Huo et al. describing severe grade thyrotoxicosis following treatment of hepatocellular carcinoma treated with chemotherapy combined with the PD-1 inhibitor tislelizumab. Symptomatic thyrotoxicosis due to thyroidits occurred after two cycles of combined treatment, complicated by rapid atrial fibrillation, resulting in dose interruption, and requring treatment with antihistamine, methimazole, and methylprednisolone. The patient became hypothyroid four-months post-thyrotoxicosis. This case is notable considering that most thyroid irAE previously reported are generally mild. Currently, there are no predictors for severe thyrotoxicosis, though there are potential candidates include pre-existing autoimmunity and thyroid autoantibodies (3).

Li et al. have presented a small case series reported PD-1-associated urethritis and cystitis, perhaps less commonly recognised than the more common nephritis. The series highlights the benefit of a detailed history for symptoms of genitourinary inflammation, simple screening to discern this from a urinary tract infection, with the benefit of avoiding unnecessary antibiotic therapy.

Together this Research Topic serves as an important contribution to the field of ICI-induced irAEs. It provides a comprehensive summary of the field to date through a bibliometric report of the topic and a deeper analysis of pancreatitis and gastrointestinal toxicity irAEs. The topic explores strategies for improved monitoring and prediction of irAEs. These include upregulated PDL-1 intra-renal and urinary kidney cells, CT radiomics, T-cell distribution and a variety of models using RNA, cellular and tumour immune correlates. This Research Topic also describes the incidence of irAEs from less well-established ICI therapies and reports of unusual irAE occurrences from standard ICI treatments. Together this diverse Research Topic synergistically melds many areas of the field in a single focus point resource.

## Author contributions

DB: Conceptualization, Writing – original draft, Writing – review & editing. MB: Conceptualization, Writing – original draft, Writing – review & editing. AM: Writing – original draft. VT: Conceptualization, Writing – original draft, Writing – review & editing. KS: Conceptualization, Writing – original draft, Writing – review & editing.
